# Risk factors associated with tendon adhesions after hand tendon repair

**DOI:** 10.3389/fsurg.2023.1121892

**Published:** 2023-04-18

**Authors:** Qiyu Jia, Dongsheng Chen, Jian Guo, Xuefeng Luo, Abudusalamu Alimujiang, Jun Zhang, Ningning Hu, Yanshi Liu, Zengru Xie, Chuang Ma

**Affiliations:** ^1^ Department of Trauma Orthopedics, The First Affiliated Hospital of Xinjiang Medical University, Urumqi, China; ^2^ Department of Orthopedics, Dingxi People's Hospital, Dingxi, China; ^3^ Department of Microrepair and Reconstruction, The First Affiliated Hospital of Xinjiang Medical University, Urumqi, China; ^4^ Department of Orthopedics, Sir Run Run Shaw Hospital, Zhejiang University, Hangzhou, China; ^5^ Department of Orthopaedics, The Affiliated Hospital of Southwest Medical University, Luzhou, China

**Keywords:** tendon adhesions, tendon repair, hand, trauma, risk facors, prevention

## Abstract

**Background:**

Tendon adhesions after hand tendon repair are one of the most difficult complications of hand surgery and can cause severe disability. This study aimed to assess the risk factors associated with tendon adhesions after hand tendon repair to provide a theoretical foundation for the early prevention of tendon adhesions in patients with tendon injuries. Moreover, this study intends to increase doctors' awareness of the issue and serves as a reference for developing new prevention and treatment strategies.

**Methods:**

We retrospectively analyzed 1,031 hand trauma cases that underwent repair after finger tendon injury in our department between June 2009 and June 2019. Tendon adhesions, tendon injury zones, and other relevant information were collected, summarized, and analyzed. The significance of data was determined using a *t*-test or Pearson's chi-square test, and odds ratios (OR) were calculated using logistic regression tests to describe factors associated with post-tendon repair adhesions.

**Results:**

A total of 1,031 patients were enrolled in this study. There were 817 males and 214 females with an average age of 34.98 (2–82) years. The injured side included 530 left and 501 right hands. Postoperative finger tendon adhesions occurred in 118 cases (11.45%), including 98 males and 20 females, 57 left and 61 right hands. The risk factors for the total sample in the descending order were degloving injury, no functional exercise, zone II flexor tendon injury, time from injury to surgery >12 h, combined vascular injury, and multiple tendon injuries. The flexor tendon sample shared the same risk factors as the total sample. Risk factors for the extensor tendon sample were degloving injury, no functional exercise.

**Conclusions:**

Clinicians should pay close attention to patients with tendon trauma in hand having the following risk factors: degloving injury, zone II flexor tendon injury, lack of functional exercise, time from injury to surgery >12 h, combined vascular injury, and multiple tendon injuries. Due to the high risk of post-repair adhesions in patients with the conditions mentioned above, individualized treatment measures should be designed for the risk factors, and postoperative functional exercise of the hand is required.

## Introduction

Bunnell stated that one of the most challenging aspects of hand surgery is the return of function in a tendon-injured finger (1).

Hand tendon injuries are a major clinical problem. Even with intricate repair, adhesion formation remains a common complication and one of the most challenging problems in hand surgery, resulting in dysfunctional finger flexion and extension (2, 3). The incidence of isolated or combined tendon injuries is approximately 30% (4). The rate of tendon adhesions after tendon injury repair is as high as 10%, with severe disability as a result, while the exact cause is unknown (5, 6). According to studies, non-operatively and surgically treated tendon injuries could be worsened by fibrotic adhesions that substantially impede hand function by interfering with the hand's gliding mechanism (5, 7). Various pathologic causes have been associated with tendon adhesions to the fibro-osseous canal and adjacent tissues (5). In addition to mechanical barriers, many pharmacologic treatments, such as hyaluronic acid, 5-fluorouracil, lubricin, and several growth factors, have been investigated for their potential to reduce adhesion formation. A hyaluronan-based gel based on an auto-crosslinked technology has been assessed in a multicenter randomized controlled trial and its effectiveness has been proven (8–13). Nonetheless, adhesions remain the most common complication that cannot be avoided. Almost 6%–20% of patients had to undergo secondary tendon adhesion release due to complications such as tendon adhesions, according to the findings of Christopher J. Dy's and numerous other studies (14, 15), causing secondary mental and physical trauma to the patients. As a result of industrialization and the harmful effects of tendon adhesions in hand, more in-depth research on hand trauma is imperative to analyze the risk factors for tendon adhesions in hand.

This study aimed to evaluate the risk factors associated with tendon adhesions after hand injury to offer a theoretical foundation for the early prevention of tendon adhesions in patients with tendon injuries, increase awareness of the issue among doctors, facilitate future research on tendon adhesions, and serve as a reference for developing new prevention and treatment strategies.

## Materials and methods

We retrospectively analyzed cases of hand trauma that underwent repair after finger tendon injury in our department between June 2009 and June 2019, following approval from our institutional review board. The inclusion criteria were: clear history of trauma such as cut, stab wound, blunt force injury, crush injury, and degloving injury; corresponding symptoms of finger tendon rupture like dysfunction of flexion and extension of the finger in the zone of the tendon innervated by the damaged tendon; concomitant or non-concomitant other injuries to the hand, for example, nerve injury, vascular injury, joint capsule and bone injury; all patients underwent surgical treatment, had no absolute contraindication to surgery; patients with complete clinical information: follow-up time >3 years; and individuals participating in the study provided informed consent. The exclusion criteria included the following: congenital finger deformities; patients with finger tendon damage due to tumor compression; patients who could not communicate due to mental abnormalities; patients with serious systemic diseases (severe cardiovascular disease, recent history of cerebral infarction, poor glycemic control of diabetes mellitus, and malignant tumors) who were incapable of tolerating surgery; patients with incomplete information; and patients with poor compliance who were unable to adhere to regular follow-up.

### Surgical procedure

The procedure was conducted under either a brachial plexus block or general anesthesia. Following a thorough debridement, the original wound of the finger grew into a jagged incision on the palmar and dorsal sides. If there was a fracture, it was first repositioned and then internally fixed. Under a microscope, the tendon sheath was exposed, and the repair was performed. The 1 cm–2 cm long tendon sheath was flapped open near the wound, and the damaged sheath was simultaneously removed; the two severed ends of the tendon were then identified for the next step of the repair. For tendon areas with sheaths, the two severed ends of the ruptured tendon were located from the sheath canal, the inactive tendonous tissue was cut, and the two severed ends of the tendon were fixed with a #5 needle to prevent tendon retraction. If both the superficial and deep flexor tendons were injured, we used to suture only the deep flexor tendon and not the superficial flexor tendon to reduce the risk of adhesions. Patients with tendon defects were treated with a tendon palmaris longus graft. First, the length of the tendon defect was measured. Next, the tendon palmaris longus of the ipsilateral forearm was taken according to the length of the defect. Finally, an appropriate length of tendon palmaris longus was grafted to the defect. If both the superficial and deep flexor tendons were simultaneously damaged, only the deep flexor tendon was grafted. The Kessler technique was used to connect the free ends of the tendon. A Tendon suture of size 3–0 was inserted longitudinally into the tendon core for a distance of 10 mm. The suture was then passed to the tendon's side, reinserted into its core, and brought back to the tendon's end. After repeating these steps on the second free tendon end, the two suture ends were connected with a surgeon's knot and three overhand knots alternating in direction. The stitches were then continued out of both tendon breaks with a 3–0 tendon suture; the tendon outer membrane was continuously sutured for one full turn, and the tendon surface was kept smooth. The injured tendon sheath was eversion sutured with a 3–0 noninvasive tendon suture, maintaining a smooth inner sheath layer. The fixed injection needle was removed, and tension testing was performed on the tendon to ensure it slid freely within the acceptable tension range. For tendon areas without sheaths, the tendon was anastomosed in the same way described above. All patients with nerve and vascular damage were microscopically healed.

### Data collection

A unified questionnaire for patients with tendon injury was designed to collect clinical data from 1,031 patients treated surgically at the First Affiliated Hospital of Xinjiang Medical University from June 2009 to June 2019. The contents of questionnaire were performed by professionally trained and qualified personnel, during which survey information and prognostic return were recorded in detail, and imperfections were followed up on time to ensure the accuracy, authenticity and reliability of the data. The clinical outcome index was the occurrence of finger tendon adhesions. The patient's gender, age, body mass index (BMI = weight (kg)/height (m^2^)), living environment (non-urban and urban), education level (below high school, high school and above), occupation (fine craftsmen and non-fine craftsmen), smoking history, drinking history, type of injury, injury cause (crush injury, cut, blunt force injury, stab wound, and degloving injury), single or multiple finger tendon injury, zone of injury (extensor tendon I–VIII zone, flexor tendon I–V zone, and multi-zone tendon injury), the season of injury occurrence (spring, summer, autumn, and winter), side (left or right), fracture (yes or no), nerve injury (yes or no), vascular injury (yes or no), time from injury to surgery (≤12 h or >12 h), postoperative functional exercise (yes or no), and the number of finger tendon adhesion cases was statistically analyzed. All included patients were at least three years after tendon repair and completed follow-up.

### Potential risk factors

The patients were divided into the tendon adhesion group (cases) and non-tendon adhesion group (cases) according to whether they had finger tendon adhesions after surgery, and the clinical data of 1,031 patients with finger tendon injuries treated by surgery were statistically analyzed. The above analysis suggests that the zone of injured extensor and flexor tendons was a risk factor for the development of tendon adhesions, necessitating a more appropriate systematization of the studied population. The clinical data of 496 patients with flexor tendon injuries and 513 patients with extensor tendon injuries were statistically analyzed, with the exception of 22 cases involving simultaneous flexor and extensor tendon injuries. Continuous variables included age and BMI. The gender, living environment (non-urban and urban), education level (below high school, high school and above), smoking history, drinking history, type of injury, injury cause (crush injury, cut, blunt force injury, stab wound, and degloving injury), single or multiple finger tendon injury, zone of injury (extensor tendon I–VIII zone, flexor tendon I–V zone, and multi-zone tendon injury), the season of injury occurrence (spring, summer, autumn, and winter), side (left or right), fracture (yes or no), nerve injury (yes or no), vascular injury (yes or no), time from injury to surgery (≤12 h or >12 h), and postoperative functional exercise (yes or no) were attributed as the categorical variables.

### Postoperative management

In patients with flexor tendon injuries, the wrist joint was cast at approximately 30° of flexion, while the metacarpophalangeal joint was cast at approximately 60° of flexion. The distal end of the cast did not extend beyond the metacarpophalangeal joint, allowing the affected finger to extend actively and flex passively within the limits of the cast. Patients with extensor tendon injuries were immobilized with a short palmar arm cast in the extended position. Active extension and passive flexion exercises were begun 24 h after surgery, 3–4 times per day with 4–6 flexions and extensions, gradually increasing to 3–5 min each time after one week, removing the external fixation and performing active extension and flexion exercises at three weeks, and beginning fist resistance training at six weeks. Patients with phalangeal fractures should be immobilized in a cast for four to six weeks before active resistance training. Strickland and Glogovac criteria and TAM method proposed by the American Society for Surgery of the Hand were used to evaluate outcomes after tendon repair (Table 1) (16).

**Table 1:** Strickland and glogovac criteria of evaluation.

Grade	Total active range of motion^a^ (degrees)	Functional return (%)
Excellent	>150	85–100
Good	125–149	70–84
Fair	90–124	50–60
Poor	<90	0–49

^a^ Sum of the active range of motion of the DIP and PIP joints.

### Statistical analysis

Statistical analysis was performed with SPSS 23.0 (IBM Corp, USA). Continuous variables, such as age and BMI, were analyzed by independent-sample *t*-tests and expressed as the mean and standard deviation. Furthermore, the categorical variables including gender, living environment (non-urban and urban), education level (below high school, high school and above), smoking history, drinking history, type of injury, injury cause (crush injury, cut, blunt force injury, stab wound, and degloving injury), single or multiple finger tendon injury, zone of injury (extensor tendon I–VIII zone, flexor tendon I–V zone, and multi-zone tendon injury), the season of injury occurrence (spring, summer, autumn, and winter), side (left or right), fracture (yes or no), nerve injury (yes or no), vascular injury (yes or no), time from injury to surgery (≤12 h or >12 h), and postoperative functional exercise (yes or no) were analyzed by the Pearson's *χ*^2^ test or Fisher exact test, expressed as the number. *P* < 0.1 was considered significant. Variables with a *P*-value of 0.1 or less were entered into a multivariate logistic regression model in a *t*-test, Pearson's *χ*^2^ test or Fisher exact test, explaining the relationship between the variables and tendon adhesions and controlling for potential confounding of any included variables. Variables with a *P*-value of 0.1 or less in the *t*-test, Pearson's *χ*^2^ test or Fisher exact test were entered into a multivariate logistic regression model to explain the relationship between variables and tendon adhesions and to control for potential confusion of any included variables. The odd ratio (OR) provided the *P*-value. A *P*-value of less than 0.05 was considered statistically significant. The cumulative risk factors were determined for each patient, and the incidence of tendon adhesions was evaluated.

## Results

A total of 1,031 patients were enrolled in this study, while 96 additional patients were lost to follow-up. There were 817 males and 214 females with an average age of 34.98 (2–82) years. The mean BMI was 23 (10–56). The injured side included 530 left and 501 right hands. There were 496 cases of flexor tendons, including 29 cases of flexor tendons with multiple zones, 513 cases of extensor tendons, including 12 cases of extensor tendons with multiple zones, and 22 cases of simultaneous injuries to flexor and extensor tendons. Postoperative finger tendon adhesions occurred in 118 cases (11.45%), including 98 males and 20 females, 57 left and 61 right hands. Of the 244 patients with combined fractures, 202 (82.79%) were fixed with Kirschner wires, of which 24 developed adhesions; 6 (2.46%) were fixed with plates, of which none developed adhesions; 4 (1.64%) were fixed with screws, of which 1 developed adhesions; 20 (8.2%) were fixed with external fixation minidevice, of which 6 developed adhesions; and 12 (4.92%) were not treated due to incomplete fractures, of which one developed adhesions. Tendon adhesiolysis was performed on 118 patients with tendon adhesions, and 116 patients were followed up for 8–30 months (mean 21 months) after surgery, with two patients lost to follow-up. Hand function was restored to more than 70% in 96 patients and less than 70% in 20 patients. There were 31 patients (3.01%) who had tendon repair due to re-rupture, and after surgery, systematic functional exercises were performed, and hand function was restored to more than 70% in 12 patients and less than 70% in 19 patients. There were 82 patients (7.95%) with joint stiffness, 14 with stiff thumb metacarpophalangeal or interphalangeal joints and 68 with stiff 2–5 fingers metacarpophalangeal or interphalangeal joints in the extension position. Patients with joint stiffness underwent lateral collateral ligament release and articular capsule release with a postoperative follow-up of 8–30 months (mean 21 months). In 37 cases, hand function was restored to greater than 70% and less than 70% in 43 cases.

Evaluation of the entire patient population. There was no significant difference in age, BMI, gender, living environment, education level, smoking history, drinking history, the season of injury occurrence, side, fracture, and nerve injury from the original cohort by the analysis of demographic data (*P *> 0.1). In contrast, occupation, open injury, injury cause, single or multiple finger tendon injury, zone of injury, vascular injury, time from injury to surgery, and postoperative functional exercise were statistically significant (*P *< 0.1). Details are given in Table 2.

**Table 2:** Univariate regression analysis of risk factors for tendon adhesions in the total sample.

Factor	Tendon adhesions	Non-tendon adhesions	*P*
Age	36.59 ± 14.95	34.78 ± 13.53	0.177
BMI	22.98 ± 3.19	22.91 ± 3.56	0.831
Gender			
Female	20	194	
Male	98	719	0.279
Living environment			
Non-Urban	39	279	
Urban	79	634	0.581
Education level			
Below high school	55	377	
High school and above	63	536	0.271
Occupation			
Non-Fine Craftsmen	85	580	
Fine Craftsmen	33	333	0.069
Smoking history			
No	70	585	
Yes	48	328	0.313
Drinking history			
No	95	764	
Yes	23	149	0.384
Opened injury			
No	5	146	
Yes	113	767	0.001
Injury cause			
Crush injury	4	138	
Cut	80	632	
Blunt force injury	18	94	
Stab wound	9	39	
Degloving injury	7	10	0.000
Single or multiple finger tendon injury			
Single tendon injury	50	554	
Multiple tendon injuries	68	359	0.000
Zone of injury			
Zone I extensor tendon	1	100	
Zone II extensor tendon	12	109	
Zone III extensor tendon	7	64	
Zone IV extensor tendon	7	59	
Zone V extensor tendon	2	24	
Zone VI extensor tendon	3	39	
Zone VII extensor tendon	2	22	
Zone VIII extensor tendon	2	48	
Zone I flexor tendon	1	34	
Zone II flexor tendon	32	96	
Zone III flexor tendon	2	46	
Zone IV flexor tendon	4	45	
Zone V flexor tendon	30	177	
Multi-zone tendon injury	13	50	0.000
Season of injury occurrence			
Spring	26	211	
Summer	42	308	
Autumn	23	172	
Winter	27	222	0.965
Side			
Left	57	473	
Right	61	440	0.474
Fracture			
No	86	701	
Yes	32	212	0.348
Nerve injury			
No	65	564	
Yes	53	349	0.161
Vascular injury			
No	87	811	
Yes	31	102	0.000
Time from injury to surgery			
≤12 h	50	473	
>12 h	68	440	0.054
Postoperative functional exercise			
Yes	75	885	
No	43	28	0.000

Factors associated with statistically significant univariate analysis were included as independent variables, and *P*-values were relaxed to < 0.1 for analysis in a binary logistic regression model. Furthermore, degloving injury, multiple tendon injuries, zone II flexor tendon injury, combined vascular injury, time from injury to surgery >12 h, and no functional exercise were significantly associated with the incidence of finger tendon adhesions. Open injuries and occupation were not significantly associated with finger tendon adhesions. Details are provided in Table 3.

**Table 3:** Multifactorial logistic regression analysis of risk factors for tendon adhesions in the total sample.

Factor	Odds ratio	*P*
Opened injury		
No	1.00	
Yes	2.086	0.223
Injury cause		
Crush injury	1.00	
Cut	1.914	0.304
Blunt force injury	3.211	0.070
Stab wound	2.421	0.248
Degloving injury	15.787	0.001
Single or multiple finger tendon injury		
Single tendon injury	1.00	
Multiple tendon injuries	1.938	0.014
Zone of finger tendon injury		
Zone I extensor tendon	1.00	
Zone II extensor tendon	6.641	0.081
Zone III extensor tendon	3.460	0.277
Zone IV extensor tendon	6.028	0.114
Zone V extensor tendon	4.407	0.247
Zone VI extensor tendon	3.380	0.325
Zone VII extensor tendon	3.036	0.406
Zone VIII extensor tendon	1.670	0.691
Zone I flexor tendon	2.348	0.559
Zone II flexor tendon	13.731	0.015
Zone III flexor tendon	1.759	0.666
Zone IV flexor tendon	3.201	0.338
Zone V flexor tendon	5.860	0.105
Multi-zone tendon injury	3.108	0.321
Vascular injury		
No	1.00	
Yes	1.974	0.025
Time from injury to surgery		
≤12 h	1.00	
>12 h	2.0	0.003
Occupation		
Non-Fine Craftsmen	1.00	
Fine Craftsmen	0.833	0.471
Postoperative functional exercise		
Yes	1.00	
No	14.108	0.000

**Figure 1:**
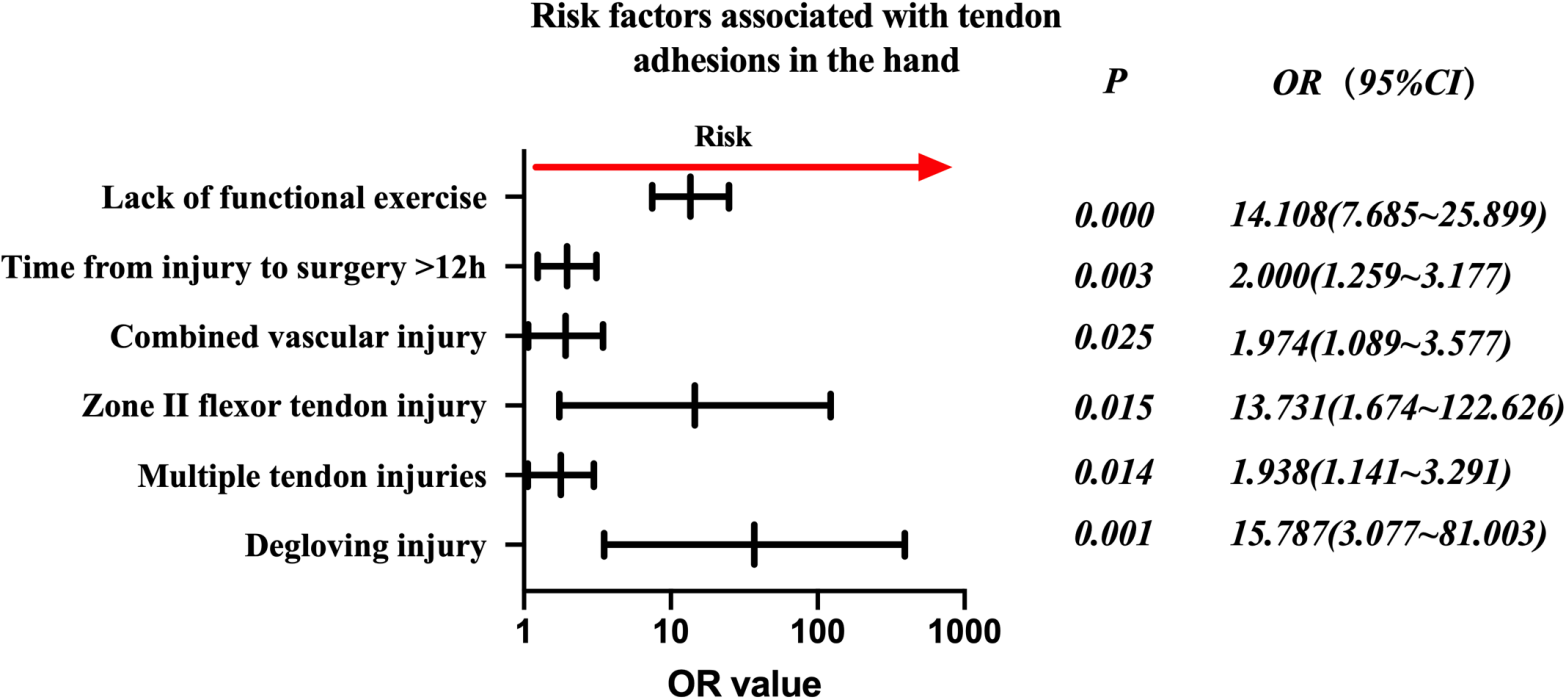
Odds ratios of risk factors associated with tendon adhesions in the hand.

The risk factors in descending order were degloving injury (*P* = 0.001, OR = 15.787, 95% CI: 3.077–81.003), no functional exercise (*P* = 0.000, OR = 14.108, 95% CI: 7.685–25.899), zone II flexor tendon injury (*P* = 0.015, OR = 13.731, 95% CI: 1.674–122.626), time from injury to surgery >12 h (*P* = 0.003, OR = 2.000, 95% CI: 1.259–3.177), combined vascular injury (*P* = 0.025, OR = 1.974, 95% CI: 1.089–3.577), and multiple tendon injuries (*P* = 0.014, OR = (1.938, 95% CI: 1.141–3.291) (Figure 1).

**Figure 2:**
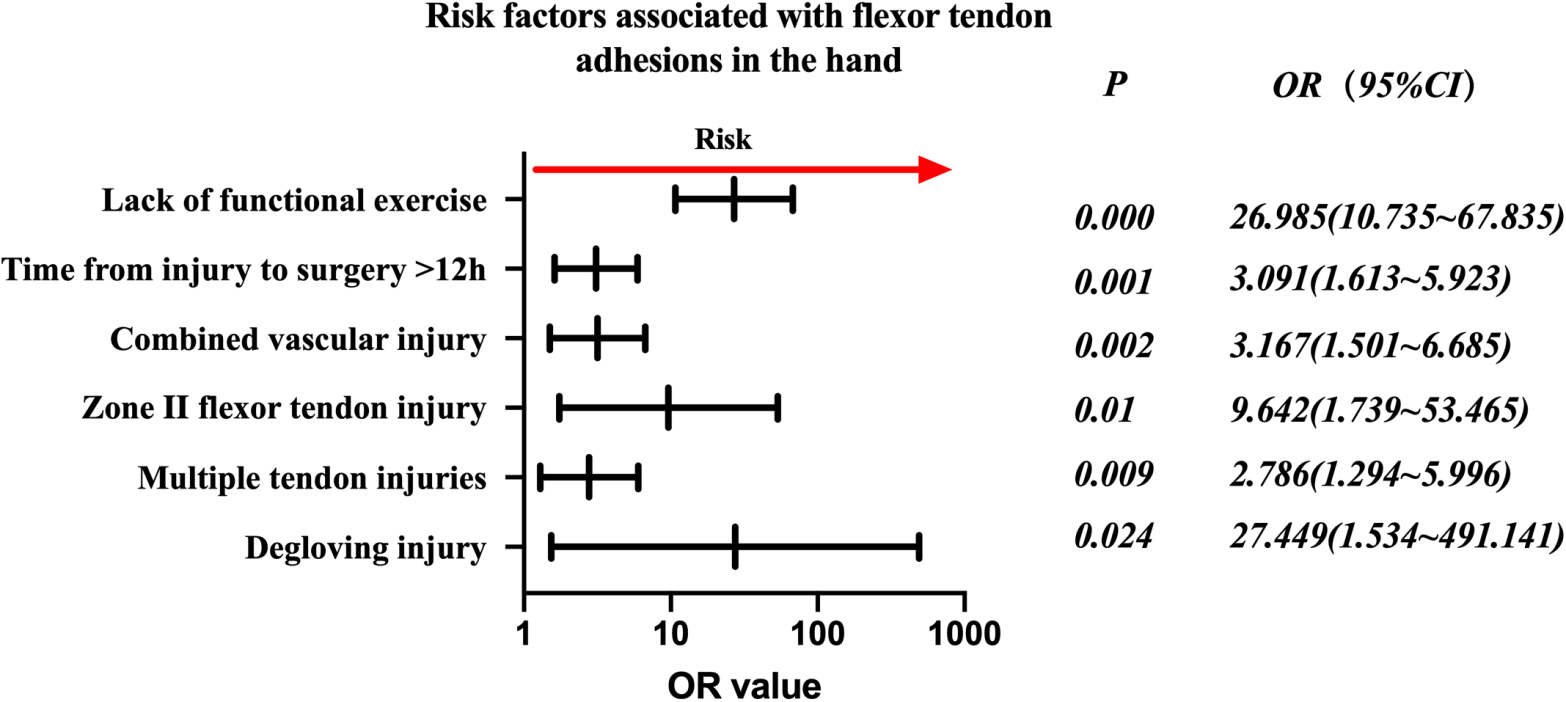
Odds ratios of risk factors associated with flexor tendon adhesions in the hand.

Injured flexor tendon patients were evaluated separately. Factors associated with statistically significant univariate analysis were included as independent variables (Table 4), and *P*-values were relaxed to <0.1 for analysis in a binary logistic regression model. Furthermore, degloving injury, multiple tendon injuries, zone II flexor tendon injury, combined vascular injury, time from injury to surgery >12 h, and no functional exercise were significantly associated with the incidence of finger tendon adhesions. Details are shown in Table 5. The risk factors in descending order were degloving injury (*P* = 0.024, OR = 27.449, 95% CI: 1.534–491.141), no functional exercise (*P* = 0.000, OR = 26.985, 95% CI: 10.735–67.835), zone II flexor tendon injury (*P* = 0.01, OR = 9.642, 95% CI: 1.739–53.465), combined vascular injury (*P* = 0.002, OR = 3.167, 95% CI: 1.501–6.685), time from injury to surgery >12 h (*P* = 0.001, OR = 3.091, 95% CI: 1.613–5.923), and multiple tendon injuries (*P* = 0.009, OR = (2.786, 95% CI: 1.294–5.996) (Figure 2).

**Figure 3:**
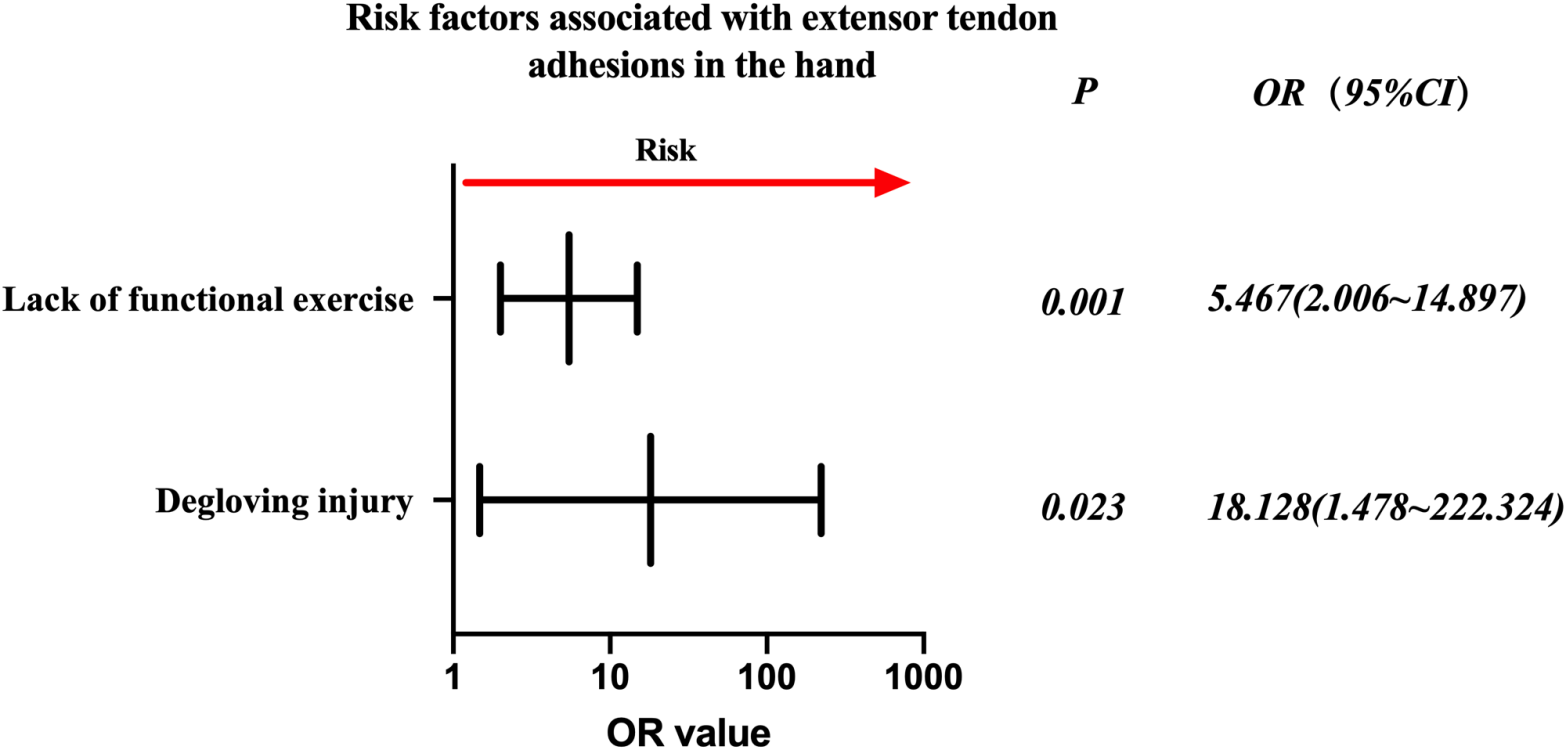
Odds ratios of risk factors associated with extensor tendon adhesions in the hand.

**Table 4:** Univariate regression analysis of risk factors for flexor tendon adhesions.

Factor	Tendon adhesions	Non-tendon adhesions	*P*
Age	34.13 ± 14.83	33.11 ± 12.90	0.535
BMI	22.47 ± 3.17	22.73 ± 3.77	0.578
Gender			
Female	13	103	
Male	63	317	0.160
Living environment			
Non-Urban	25	130	
Urban	51	290	0.737
Education level			
Below high school	22	171	
High school and above	44	249	0.820
Occupation			
Non-Fine Craftsmen	51	266	
Fine Craftsmen	25	154	0.529
Smoking history			
No	43	263	
Yes	33	157	0.319
Drinking history			
No	59	339	
Yes	17	81	0.535
Opened injury			
No	2	21	
Yes	74	399	0.555
Injury cause			
Stab wound	3	19	
Cut	55	346	
Blunt force injury	11	32	
Crush injury	4	21	
Degloving injury	3	2	0.026
Single or multiple finger tendon injury			
Single tendon injury	24	210	
Multiple tendon injuries	52	210	0.003
Zone of injury			
Zone I flexor tendon	1	34	
Zone II flexor tendon	32	96	
Zone III flexor tendon	2	46	
Zone IV flexor tendon	4	45	
Zone V flexor tendon	30	177	
Multi-zone tendon injury	7	22	0.001
Season of injury occurrence			
Spring	20	105	
Summer	25	155	
Autumn	14	66	
Winter	17	94	0.891
Side			
Left	32	196	
Right	44	224	0.463
Fracture			
No	63	353	
Yes	13	67	0.801
Nerve injury			
No	29	184	
Yes	47	236	0.360
Vascular injury			
No	51	369	
Yes	25	51	0.000
Time from injury to surgery			
≤12 h	28	255	
>12 h	48	165	0.000
Postoperative functional exercise			
Yes	44	412	
No	32	8	0.000

**Table 5:** Multifactorial logistic regression analysis of risk factors for flexor tendon adhesions.

Factor	Odds ratio	*P*
Injury cause		
Stab wound	1.00	
Cut	2.812	0.245
Blunt force injury	5.66	0.08
Crush injury	3.624	0.255
Degloving injury	27.449	0.024
Single or multiple finger tendon injury		
Single tendon injury	1.00	
Multiple tendon injuries	2.786	0.009
Zone of finger tendon injury		
Zone I flexor tendon	1.649	0.71
Zone II flexor tendon	9.642	0.01
Zone III flexor tendon	1.00	
Zone IV flexor tendon	1.744	0.604
Zone V flexor tendon	3.424	0.158
Multi-zone tendon injury	1.592	0.668
Vascular injury		
No	1.00	
Yes	3.167	0.002
Time from injury to surgery		
≤12 h	1.00	
>12 h	3.091	0.001
Postoperative functional exercise		
Yes	1.00	
No	26.985	0.000

Injured extensor tendon patients were evaluated separately. Factors associated with statistically significant univariate analysis were included as independent variables (Table 6), and *P*-values were relaxed to <0.1 for analysis in a binary logistic regression model. Furthermore, degloving injury and no functional exercise were significantly associated with the incidence of finger tendon adhesions. Details are shown in Table 7. The risk factors in descending order were degloving injury (*P* = 0.023, OR = 18.128, 95% CI: 1.478–222.324), no functional exercise (*P* = 0.001, OR = 5.467, 95% CI: 2.006–14.897) (Figure 3).

**Table 6:** Univariate regression analysis of risk factors for extensor tendon adhesions.

Factor	Tendon adhesions	Non-tendon adhesions	*P*
Age	40.74 ± 14.69	36.13 ± 13.89	0.051
BMI	23.92 ± 3.14	23.08 ± 3.38	0.141
Gender			
Female	6	90	
Male	32	385	0.631
Living environment			
Non-Urban	12	141	
Urban	26	334	0.806
Education level			
Below high school	19	201	
High school and above	19	274	0.357
Occupation			
Non-Fine Craftsmen	30	302	
Fine Craftsmen	8	173	0.056
Smoking history			
No	25	311	
Yes	13	164	0.969
Drinking history			
No	32	409	
Yes	6	66	0.746
Opened injury			
No	3	123	
Yes	35	352	0.013
Injury cause			
Crush injury	2	113	
Cut	24	275	
Blunt force injury	6	61	
Stab wound	4	19	
Degloving injury	2	7	0.008
Single or multiple finger tendon injury			
Single tendon injury	23	333	
Multiple tendon injuries	15	142	0.218
Zone of injury			
Zone I extensor tendon	1	100	
Zone II extensor tendon	12	109	
Zone III extensor tendon	7	64	
Zone IV extensor tendon	7	59	
Zone V extensor tendon	2	24	
Zone VI extensor tendon	3	39	
Zone VII extensor tendon	2	22	
Zone VIII extensor tendon	2	48	
Multi-zone tendon injury	2	10	0.059
Season of injury occurrence			
Spring	6	106	
Summer	16	149	
Autumn	7	101	
Winter	9	124	0.576
Side			
Left	25	267	
Right	13	208	0.251
Fracture			
No	23	340	
Yes	15	135	0.149
Nerve injury			
No	34	372	
Yes	4	103	0.103
Vascular injury			
No	34	434	
Yes	4	41	0.691
Time from injury to surgery			
≤12 h	18	208	
>12 h	20	267	0.669
Postoperative functional exercise			
Yes	30	456	
No	8	19	0.000

**Table 7:** Multifactorial logistic regression analysis of risk factors for extensor tendon adhesions.

Factor	Odds ratio	*P*
Age	1.022	0.096
Opened injury		
No	1.00	
Yes	2.015	0.411
Injury cause		
Crush injury	1.00	
Cut	2.368	0.337
Blunt force injury	3.082	0.211
Stab wound	5.611	0.104
Degloving injury	18.128	0.023
Occupation		
Fine Craftsmen	1.00	
Non-Fine Craftsmen	1.539	0.342
Zone of finger tendon injury		
Zone I extensor tendon	1.00	
Zone II extensor tendon	5.615	0.112
Zone III extensor tendon	4.94	0.156
Zone IV extensor tendon	5.516	0.13
Zone V extensor tendon	3.836	0.294
Zone VI extensor tendon	3.288	0.329
Zone VII extensor tendon	3.176	0.385
Zone VIII extensor tendon	1.762	0.657
Multi-zone tendon injury	3.667	0.394
Postoperative functional exercise		
Yes	1.00	
No	5.467	0.001

## Discussion

Adhesion formation after tendon repair remains challenging despite advances in surgical techniques and postoperative rehabilitation protocols for the hand. The present study reported a high tendon adhesion rate of 11.45% among 1,031 patients with finger tendon injuries, slightly higher than previous studies (5, 6). This shows that the rate of tendon adhesions has remained high and plagued numerous surgeons and patients to date. The logistic regression analysis of the total sample demonstrated that degloving injury, multiple tendon injuries, zone II flexor tendon injury, combined vascular injury, time from injury to surgery >12 h, and no functional exercise had significant and independent negative impacts on reoperation. The open injuries and occupation were univariately associated with reoperation but did not significantly contribute to the regression model. Moreover, age, BMI, gender, living environment, education level, smoking history, history of alcohol consumption, the season of injury, laterality, fracture, nerve injury, and some previously identified factors that might contribute to the risk of tendon adhesions, did not differ between groups (14, 17).

Consistent with previous studies (18, 19), the findings of this study established that the majority of patients with tendon injuries were young (mean age 34.98 years), male (79.24%), and blue-collar workers (52.47%). In addition, 35.5% of the sample had fine jobs that relied on flexible hand functions, such as computer operators and handicraft makers. Subsequently, appropriate impairment management is crucial to optimize long-term functional outcomes for such patients. Adequate hand function increases the working life expectancy of these patients (20). However, a prognosis based on tendon adhesion risk factors is critical in improving the hand function of patients.

Skin avulsion from the underlying structures usually results from trauma. A degloving injury attains more significance in hand because of the irreplaceable quality of the skin that has been lost and the exposure of delicate structures of the hand (21); results of this study revealed that once the injury is complicated by tendon, it is highly susceptible to tendon adhesions at a later stage and this risk factor had the highest OR in our study. Caliskan Uckun A et al. (17) found that the larger the Modified Hand Injury Severity Scoring (MHISS) (22), the poorer the functional recovery of the hand after tendon injury repair. Degloving injury resulting in large MHISS values, irregular wound trauma, and severe wound contamination might increase edema, pain, and subsequent fibrosis and could be combined with multiple nerves, vascular, and fracture injuries that severely disrupt the blood supply to tendon cells, leading to an increased risk of complications and associated reoperation.

Zone II injury of the flexor tendon is a well-known risk factor for adverse outcomes (23–25); due to the complex anatomy of this zone, containing the flexor digitorum superficialis (FDS) and flexor digitorum profundus (FDP) within its narrow tendon sheath (26). Therefore, we discovered that zone II flexor tendon injury was associated with tendon adhesions compared to other zones. This was one of the important factors that prompted us to further systematize the population, grouping and refining the study based on whether the injury was to a flexor or extensor tendon. Zone II flexor tendon is also referred to as “no-man's-land” (27), where FDS and FDP are confined within the narrow tendon sheath, and outside the tendon sheath, there are five annular, four cruciform and one palmar aponeurosis pulleys, which play a crucial role in tendon gliding. Once the injury occurs in this zone, it is very easy for the sheath and pulley to sustain combined damage. FDS and FDP rupture simultaneously in the narrow sheath, destroying the physiological anatomy of double tendons in zone II and causing FDS to lose its good sliding base bed. Simultaneously, the inflammatory granulation tissue produced by the ends of FDS and FDP easily causes adhesions in the tendon sheath, restricting the sliding of FDS and FDP within the tendon sheath. Furthermore, the tendon has a poor nutritional supply in this zone, and the injury to the pulleys and aponeurosis significantly impact the tendon's nutritional supply; consequently, adhesions are very easy to form in the pulley area after a tendon injury in zone II. According to the results of the total sample analysis, OR for the flexor tendon zone II injury risk factor was 13.731. This result suggested that hand surgeons should pay close attention to patients who have sustained injuries in this zone. The tendon rupture should be repaired using microscopic techniques for a flat and smooth repair. If the sheath and pulley are both damaged, the repair should be performed concurrently, restoring the anatomical function of the sheath and pulley, and the pulley should be reconstructed if it is severely damaged. It should be verified that the tendon repair is encased within the tendon sheath. If the tendon sheath is embedded in the tendon break, it might be necessary to partially remove the embedded tendon sheath and reconstruct the function of the tendon sheath in zone II to avoid affecting the slide of the flexor tendon in the tendon sheath (28). If FDS and FDP rupture simultaneously, they should be repaired concurrently if the tension of the tendon permits (24). The blood supply to the vincula of tendons and paratendon should be restored by microsurgical techniques to repair the blood vessels and tendon sheaths, and to restore the physiological and anatomical function of both tendons in the tendon sheath of zone II should be restored, enhancing the biomechanical strength of the tendon, providing the biomechanical guarantee of the tendon for early postoperative functional exercise, and preventing tendon adhesions.

Multiple tendon injuries have been linked to poor functional recovery following tendon repair, as reported by Rigo and Elhassan (23). This finding echoed in this investigation, where patients with multiple flexor tendon injuries were more likely to suffer tendon adhesions. Patients with multiple tendon injuries have a high degree of trauma, and during the inflammatory phase of tendon healing (48–72 h), more inflammatory granulation tissue is produced at the severed ends of multiple tendons compared to patients with single tendon injuries, resulting in several fibroblasts in the tendon's outer membrane embedded in its granulation tissue surface during the fibroblastic phase of tendon healing (five days to four weeks), while continuously proliferating and accumulating collagen to form more collagen fibers. Excessive collagen will cause tendon adhesions, and multiple tendon injuries will inevitably result in excessive scarring, which will cause tendon adhesions and limit tendon slipping, further reducing tendon function and leading to chronic complications (29, 30). The finding revealed that in patients with multiple tendon injuries, the tendon-severed ends should be repaired with microscopic techniques to make each severed end of the tendon flat and smooth while ensuring thorough debridement (31). Smoothness of the tendon severed ends should be ensured to prevent the tendon from being caught in the tendon sheath during sliding, which can lead to tendonitis or limited flexion and extension and prevent secondary tendon injury during suturing. Furthermore, a suitable and correct number of suture strands should be chosen to ensure a good tendon suture structure and to improve tendon biomechanical strength. The details of suturing process should be handled properly using microsurgical techniques; clamping the tendon should be gentle, avoiding excessive and unnecessary clamping to reduce the production of collagen fibers during the tendon healing process, inhibiting the exogenous healing process of the tendon, and reducing the occurrence of tendon adhesions.

Tendon repair surgery should focus on reconstructing the tendon's fundamental structures, particularly the tendon's nutrition and blood supply. The findings of this study verified the existence of combined vascular injury as a risk factor for the development of flexor tendon adhesions. Compared to many other tissues, tendons are hypovascular, and the hand tendons are believed to have a more limited vascular supply (32–34). Nonetheless, synovial fluid can make up for the disparity in vascular supply (8). In the short term, peritendinous tissue blood flow and anatomy must be re-established to meet the nutritional requirements for tendon healing, whether by blood supply or synovial fluid infiltration. Allowing endogenous tendon healing to predominate has a beneficial impact on adhesion prevention following tendon rupture suturing.

This study found that a delay of >12 h between injury and surgery increased the risk of flexor tendon adhesions (*P *< 0.05). This is because the delay between injury and surgery causes prolonged ischemia of the tendon and the invasion of inflammatory tissue into the tendon and surrounding tissues, increasing the risk and severity of the infection and severe retraction of the tendon's severed end, causing increased tension around the tendon and allowing a gap to form around the tendon (24, 35, 36). These gaps serve as a breeding ground for infected lesions and inflammatory tissues, which cause edema of the tendon severed ends and eventually lead to the formation of adhesions during the fibroblastic phase of tendon healing (37, 38). Consequently, we should alert such individuals to the dangers of tendon injury, particularly by educating patients in remote areas about safety, popularizing the general knowledge of rescue and treatment after hand trauma to reduce the incidence of tendon adhesions by seeking prompt medical attention in the event of a hand injury, early debridement and treatment, and phase I or II repair, as appropriate.

The OR for the total sample of patients who did not perform functional exercise was 14.108. Although this result was not unsurprising, there is no doubt that the proportion of patients who did not perform functional exercise among patients with complications of tendon adhesions is very high. A significant proportion of patients were afraid to initiate functional exercise due to postoperative pain and the psychological barrier of fear of re-rupture; hence, they missed the optimal recovery period. In this regard, postoperative education and guidance in rehabilitation exercises are especially important for these patients. Most scholars have agreed on the early postoperative protective active and passive exercise. Moreover, the repeated sliding of the tendon can prevent the peritendinous fibroblasts from growing into the tendon rupture. Durbert (39) advised early active functional exercise to reduce the occurrence of tendon adhesions by blocking the long-term contact between tendon and scar tissue through repeated sliding of the tendon, inhibiting the exogenous healing process of the tendon. Functional exercise should be balanced with tendon protection. Within two weeks after surgery, passive functional exercise should follow the principle of slow and adequate amplitude to avoid tendon re-rupture. Active functional exercise should be performed about four weeks after surgery. Even so, tendon adhesions still occur from time to time and are unpredictable (40, 41). Duzgun I et al. (42) considered around five days postoperatively as the best time to start the passive activity. However, good suturing techniques and early endogenous healing are the basis for early activity. Atik et al. (43) invented the angular technique of interlocking, which was validated in an animal model and concluded that tendon repair using this suture method has a higher fibroblast and collagen content than the modified Kessler technique and is more conducive to early rehabilitation to prevent adhesion formation. Contrary to our initial assumptions, the findings indicate that fractures are not a risk factor. This could be attributed to the impact of fracture patients being subjected to more passive interviews and educations as a result of the need for routine post-operative x-rays. Thus, these patients were typically more aware of post-operative exercise. Patients with fractures were more likely to believe that they would experience difficulty moving their fingers later in life than patients with non-combined fractures. Patients with fractures were consequently more likely to adhere to exercise. In the future, additional in-depth research on this topic is required.

Further analyses in this study suggested that grouping based on the zone of injury and whether it was a flexor or extensor tendon was necessary, and that the zone of injury was correlated with the possibility of adhesion development. The flexor tendon sample (Figure 2) shared the same risk factors as the total sample (Figure 1). Extensor tendon risk factors included degloving injury and lack of functional exercise (Figure 3), but this does not mean that the three factors of time from injury to surgery >12 h, combined vascular injury, and multiple tendon injuries do not need to be considered in the case of a simple extensor tendon injury. The recovery of a patient's hand function is heavily dependent on the decisions of the treating surgeon regarding the severity of the hand injury, the timing of surgery, the management of comorbidities, and postoperative rehabilitation. A skilled surgeon must perform a successful repair and provide the patient with ongoing postoperative care and guidance. A personalized risk assessment and prognostic evaluation of the patient's injury status before surgery could significantly impact the outcome. This should be followed by individualized treatment, as mentioned previously, with particularly delicate sutures for patients with risk factors and psychological support if the patient has a fear of exercise as a psychological barrier.

There were several limitations in this study. First, some patients with skin defects would undergo flap grafting, which would inevitably affect the incidence of tendon adhesions in the future. However, the patients who received flap grafts had various surgical procedures and a relatively high degree of variability, which was excluded from the statistics because it would have increased the degree of variability in the model. Future research should focus on this aspect in greater detail. Second, the necessity of postoperative smoking cessation was heavily emphasized to our patients at the time of admission, and most patients were willing to cooperate, based on the severity of smoking in this type of disease, as demonstrated by previous research. For the sake of analytical rigor, however, only previous smoking history was included in the study, and postoperative smoking was not analyzed. During follow-up, a certain percentage of patients provided false information about smoking cessation, inconsistent with the information provided by their families out of fear of reprimand from their physicians. This section will be a major focus of our future research. Lastly, the sample size of this study was adequate, but it was a single-center study that could have biased the results, and additional multicenter studies must be conducted in the future.

## Conclusion

When patients with tendon trauma in hand have the risk factors of degloving injury, lack of functional exercise, zone II flexor tendon injury, time from injury to surgery >12 h, combined vascular injury, and multiple tendon injuries, clinicians must pay close attention. Given the possibility of a high risk of post-repair adhesions in patients with the aforementioned conditions, individualized treatment measures should be designed for the risk factors, and postoperative functional exercise of the hand is required.

## Data Availability

The raw data supporting the conclusions of this article will be made available by the authors, without undue reservation.
